# Supplementary material for: DMRforPairs: identifying Differentially Methylated Regions between unique samples using array based methylation profiles
Source: BMC Bioinformatics. 2014 May 15;15:141. doi: 10.1186/1471-2105-15-141 (PMC4046028; doi:10.1186/1471-2105-15-141)
Supplement: Additional file 2 — DMRforPairs output for the comparison of A431-MCF7 and NA17018-NA17105. Please start from the HTML files in each folder. Available via the BMC Bioinformatics website. [file 1471-2105-15-141-S2.zip › 1394847754114233_MOESM2_ESM/A431_MCF7/figures/17326.pdf]

RegionID: 17326, chr17:38334161−38334186−M_values

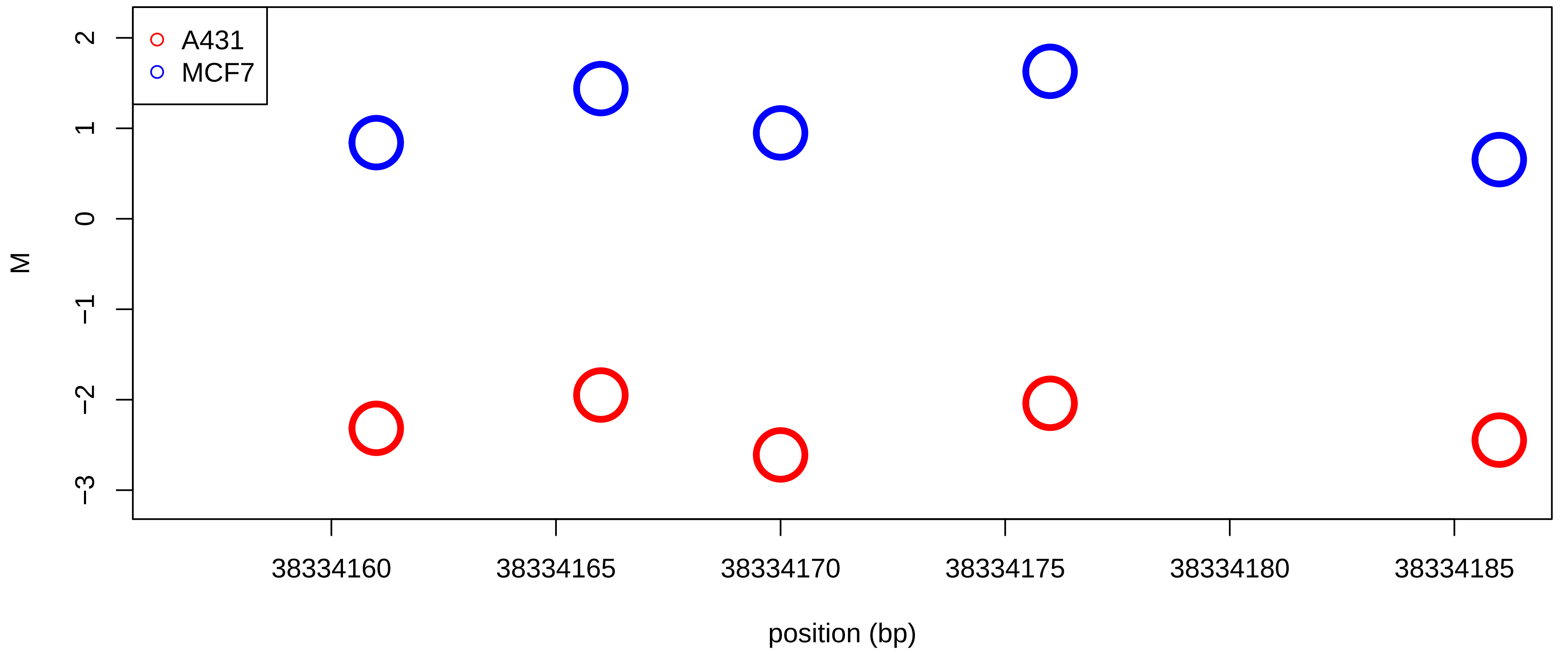

RegionID: 17326, chr17:38334161−38334186−Beta_values

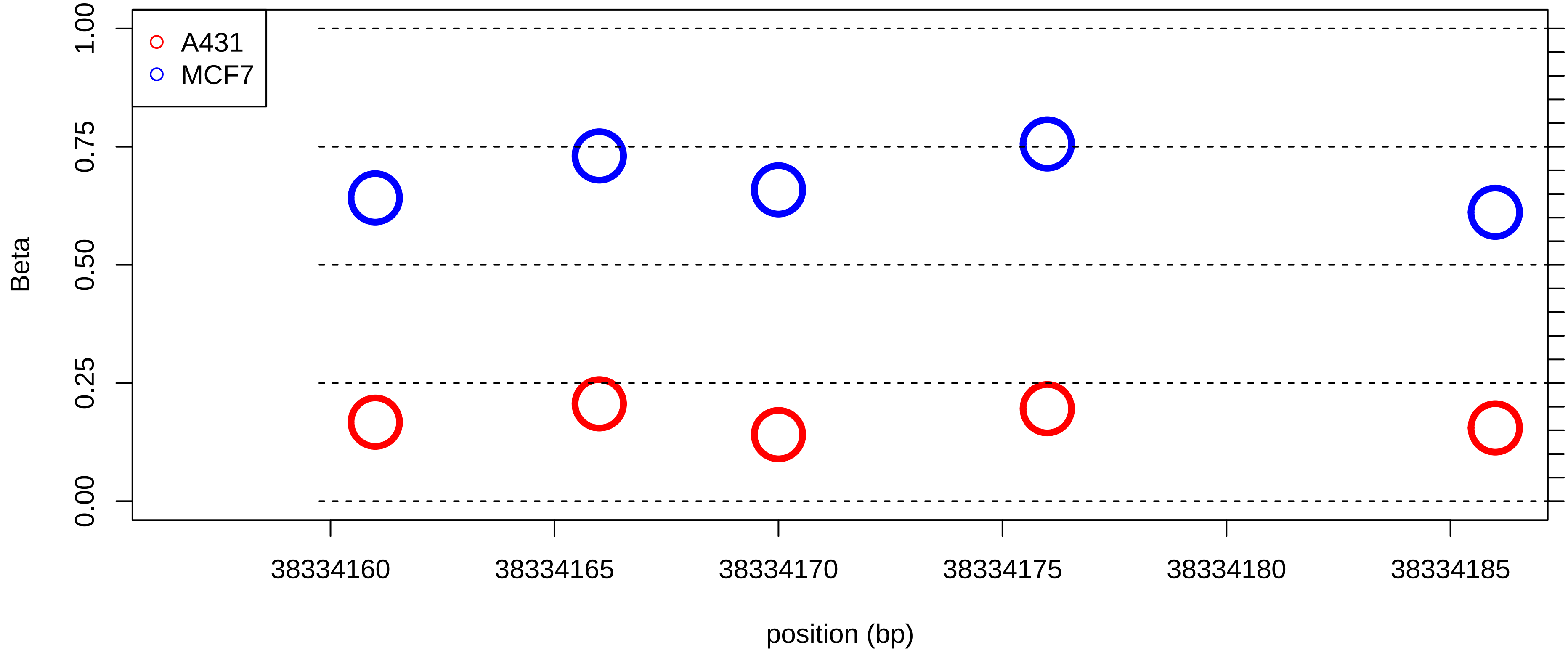

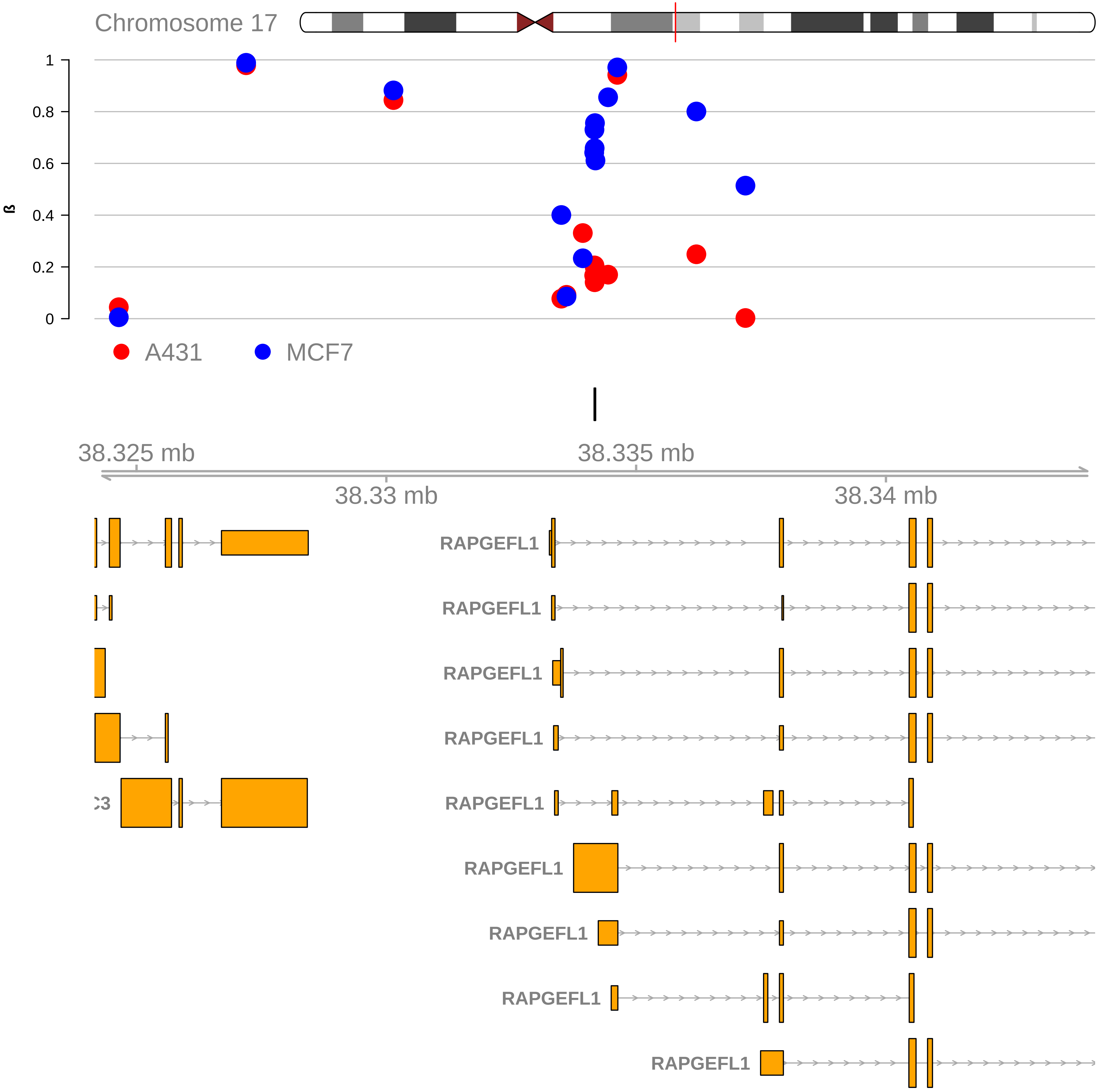

Chromosome 17
1
0.8
0.6
0.4
0.2
0
ß
A431
MCF7
38.325 mb
38.335 mb
38.33 mb
38.34 mb
RAPGEFL1
RAPGEFL1
RAPGEFL1
RAPGEFL1
RAPGEFL1
RAPGEFL1
RAPGEFL1
RAPGEFL1
RAPGEFL1
